# The Impact of Globalization on Forest Growth: Evidence from Multinational Panel Data

**DOI:** 10.3390/ijerph182412969

**Published:** 2021-12-08

**Authors:** Quan-Jing Wang, Yong Geng, Xi-Qiang Xia

**Affiliations:** 1 School of Business, Zhengzhou University, 100 Kexue Road, Zhengzhou 450001, China; wqj@zzu.edu.cn; 2 School of Environment and Science Engineering, Shanghai Jiaotong University, 800 Dongchuan Road, Shanghai 200240, China; ygeng@sjtu.edu.cn

**Keywords:** forest growth, globalization, moderating effect, different political regime, multinational research

## Abstract

Even though some existing literature has studied the impact of globalization on forest growth, this research remains inconclusive; moreover, little clarification has emerged as to whether the influence of globalization on forest growth is consistent among different countries. To fill this research gap, we investigated the impact of globalization on forest growth and considered what factors could change the influence of the former upon the latter. To empirically investigate these essential issues, we utilized cross-country data covering 108 countries during the period 1991–2018 to conduct a system generalized method of moment (GMM) estimation. The baseline results confirm the positive impact of globalization on forest growth, which is also supported by several robustness tests, such as changing the measurements and setting new samples. Furthermore, an increase in globalization would bring about higher forest growth. Aside from this, two specific dimensions of globalization, namely economics and trade, can also protect forest growth. Additionally, a higher FDI strengthens the positive impact of globalization on forest growth, while aging, industrial share, and CO_2_ emissions weaken it. Finally, the impact of globalization on forests is weaker in democracies, emerging markets, and countries with higher fiscal freedoms, while it is stronger in countries with higher political stability. Our study provides substantial policy implications for governments participating in international treaties related to forest growth. The structure of this paper is organized as follows.

## 1. Introduction

In recent decades, the world economy has experienced rapid growth; however, at the same time, forests have been ignored and deforestation has grown. Since the carbon emissions caused by deforestation cause problems such as global warming, human diseases, and the extinction of large numbers of species (Strassburg et al., 2012; Roopsind et al., 2019) [1,2], the protection of forests remains essential to ensure environmental quality, sustainable social development, and biodiversity (Nanni et al., 2019; Sloan, 2022) [3,4]. Therefore, it is necessary for governments to take measures to protect forests. In this vein, many scholars have investigated the notion of forest growth from the perspectives of corruption, infrastructure, urban expansion, agriculture food, etc. (Hosonuma et al., 2012; Shi et al., 2017) [5,6]. However, among the existing literature, few studies have examined globalization’s direct impact on forest growth, and no studies have investigated what factors could influence the impact of the former upon the latter.

There are two opposing ideas regarding the role of globalization in forest growth. One strand of literature suggests that globalization harms forest growth. For instance, Meyfroidt et al. (2010) [7] state that economic globalization may bring about the displacement of deforestation overseas; this can facilitate forest transitions on a national scale but reduce the regional or global environmental benefits of policies related to forest growth. Rudel (2002) [8] also state that globalization may destroy primary forests in some areas. Similarly, Ramsfield et al. (2016) [9] suggest that natural forest ecosystems will experience continuing threats caused by globalization. Aide and Grau (2004) [10] argue that, even though the globalization process does some good to high-yield agriculture—as it increases market demand and decreases cost, as well as improving agricultural production through technology—it ignores many environmental issues such as land use and forests due to the expansion of agricultural production activities that require more land, eventually causing greater deforestation (Grau and Aide, 2008) [11].

Other researchers argue that globalization benefits forest growth. For instance, Daniels (2009) [12] argues that globalization causes a decline in beef prices, which eventually positively impacts forest cover in Costa Rica due to the concomitant reduction in animal husbandry. Meyfroidt and Lambin (2009) [13] argue that current forest recovery in several Latin American countries was caused by a reduction in domestic agricultural production heralded by international trade. Li et al. (2017b) [14] studied the influence of economic globalization by empirically examining the impact of trade on forest transition via data for 76 developing countries. They concluded that, if other factors remain unchanged, net import of wood would reduce deforestation activities. Hecht et al. (2006) [15] proposed that, while globalization causes deforestation in many regions in Latin America, it also causes greater forest resurgence, which can lead to more forest growth. For example, the recuperation of forests in EI Salvador was an outcome of economic and political globalization. Similarly, some studies propose that globalization is highly related to forest management, as it affects land use policy, as well as non-governmental organizations (NGOs) and conventions (Meyfroidt and Lambin,2011; Hecht, 2010) [16,17], Grainger (2005) [18]). In addition, Rudel (2002) [8] stated that globalization does some good to the secondary growth of forest in some places.

By browsing the existing literature, it is easy to find that previous studies attributed more importance to drivers of forest deforestation and forest growth; however, these conclusions are controversial, and studies that empirically test the direct influence of globalization on forest growth, as well as those that consider whether the impact of globalization on forest growth is consistent among different countries, are rare.

According to a review of the relevant literature and a theoretical analysis of the impact of globalization on forest growth, this paper uncovers the relationship between globalization and forest growth and examines whether the impact of globalization on forest growth varies among different countries; as such, it fills the existing research gap related to the relationship between globalization and forest growth. Based on this approach, we propose the following questions: Does the overall or specific aspect of globalization exert a significant impact on forest growth? Is the impact of globalization on forest growth consistent among different countries?

Globalization may affect forest growth through the following transmission channels. First, according to the traditional theory of international trade, globalization usually causes three effects: a scale effect, a composition effect, and a technology effect (Copeland and Taylor, 2013) [19]; these may change economic activities and production methods and eventually affect the utilization of land or forests. However, as far as we are concerned, globalization not only means economic globalization or trade openness but represents a multifaceted phenomenon with essential social and political dimensions, as the environmental problem is a worldwide issue on which nearly all countries agree (Yang et al., 2021) [20]; thus, the deforestation caused by the scale effect of globalization may be somewhat weakened (Li et al., 2017a) [21], meaning that international trade or investment is more likely to present a technological effect for improving production methods in an environmentally friendly manner (Wang et al., 2021b) [22].

Second, Lambin and Meyfroidt (2011) [23] propose that the forest transition should be linked to the transfer or replacement of deforestation activities, i.e., globalization can bring about replacements overseas, which may promote national forest growth by reducing domestic deforestation. Furthermore, globalization changes food generation and supply by international trade, reduces domestic agriculture production and eventually stimulates the forest resurgence (Grau et al., 2013) [24]. Moreover, globalization is critical for building and spreading international networks, which can eventually promote forest growth through environmental globalization (Grainger, 2005) [18]. In addition, Kull et al. (2007) [25] suggested that the labor migration led by globalization could cause a greater remittance (the widespread transfer of money from foreign workers to their home countries) flow between sending and receiving countries, while Hecht et al. (2006) [15] indicated that high remittances are positively correlated with forest recovery. Hence, international tourists and real estate investors created by globalization would affect local economic diversification, such as improving non-farming activities (Stem et al. 2003) [26], which may lead to more forest cover (Dib et al., 2018) [27]. Aside from these notions, globalization does some good in spreading sustainable development and ecosystem protection, leading to more aid agencies, international NGOs, researchers, and communities that aim to protect forests across national borders.

However, the impact of globalization on forest growth may vary among different countries or regions. Take the Amazonian forests as an example. Since Amazonian deforestation has reached a historic level (Yanai et al., 2020; Maeda et al., 2021) [28,29], the impact of globalization on deforestation is somewhat weak, even imperceptible; thus, the impact of globalization on forest growth in such areas is different than in other regions. Moreover, the characteristics of social structure, political structure, and environmental quality also change the attitudes of governments and individuals to natural environments and forests (Wang et al., 2021b) [22]; this change may, in turn, affect the impact of globalization on forest growth. Based on such a theoretical analysis, we propose two hypotheses:

**Hypothesis** **1.** *Globalization benefits national forest growth*.

**Hypothesis** **2.** *The impact of globalization on forest growth varies among different countries, and it depends on the social, political, and geographical features of each country*.

The potential contributions of this work are as follows. First, contrary to previous literature investigating the relationship between globalization and the environment from the perspective of carbon emissions or ecosystems, we focus on the relationship between globalization and forest growth, which counterbalances the dearth of existing literature, as forests contribute to a significant share of the total environment (Li et al., 2017a) [21]. Next, previous studies focused on forests often utilize data for one or a few countries to conduct empirical investigations based on static ordinary least squares estimations, which may create biased conclusions (Harper et al., 2007; Van Khuc et al., 2018; Vergara-Asenjo and Potvin, 2014) [30,31,32]. Our study differs insofar as we conduct a system generalized method of moment (GMM) estimation, which allows for the dynamic progress of forest growth based on cross-country data from 108 countries to gain a widely accepted and credible conclusion on the relationship between globalization and forest growth (Li et al., 2017b) [14]. Hence, with the growth of protectionism, we move a step further to examine whether the increase or decrease in globalization affects forest growth; this is a novel approach not seen in previous studies (Wang et al., 2021b) [22].

Additionally, we study whether the globalization’s impact on forest growth is consistent among countries with different levels of aging, FDI, industrial share, and air quality, as well as between equatorial countries and non-equatorial countries and emerging markets and non-emerging markets; this approach links globalization to social indicators and better clarifies the relationship between globalization and forest growth in fundamental societies (Aldyan, 2020) [33]. Finally, we take different political regimes into account, such as democracies, left-wing countries, as well as countries with different levels of freedom or political stability, to comprehensively capture the differences related to globalization’s impact on forest growth (Kull et al., 2007) [25].

## 2. Materials and Methods

To answer above questions, we carry out an empirical investigation by employing multinational data covering 108 countries during the period 1991–2018, via the system GMM estimation. We utilize net forest growth to measure forest growth and capture globalization by the KOF index provided by Dreher (2006) [34].

### 2.1. Variables and Data

Forest growth (forest): since there are large differences in forest endowment among countries, potential biases might emerge if were we to measure forests by their absolute area. According to the previous literature (Agarwal, 2009) [35], we utilized the net change in forest by the total area of forest to capture forest growth (hereafter forest). If forest is larger than 0, this implies that the forest is regrowing more than it is being lost.

**Figure 1:**
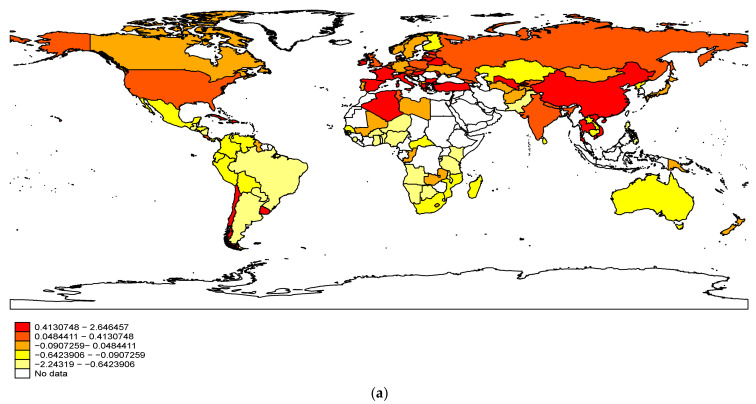

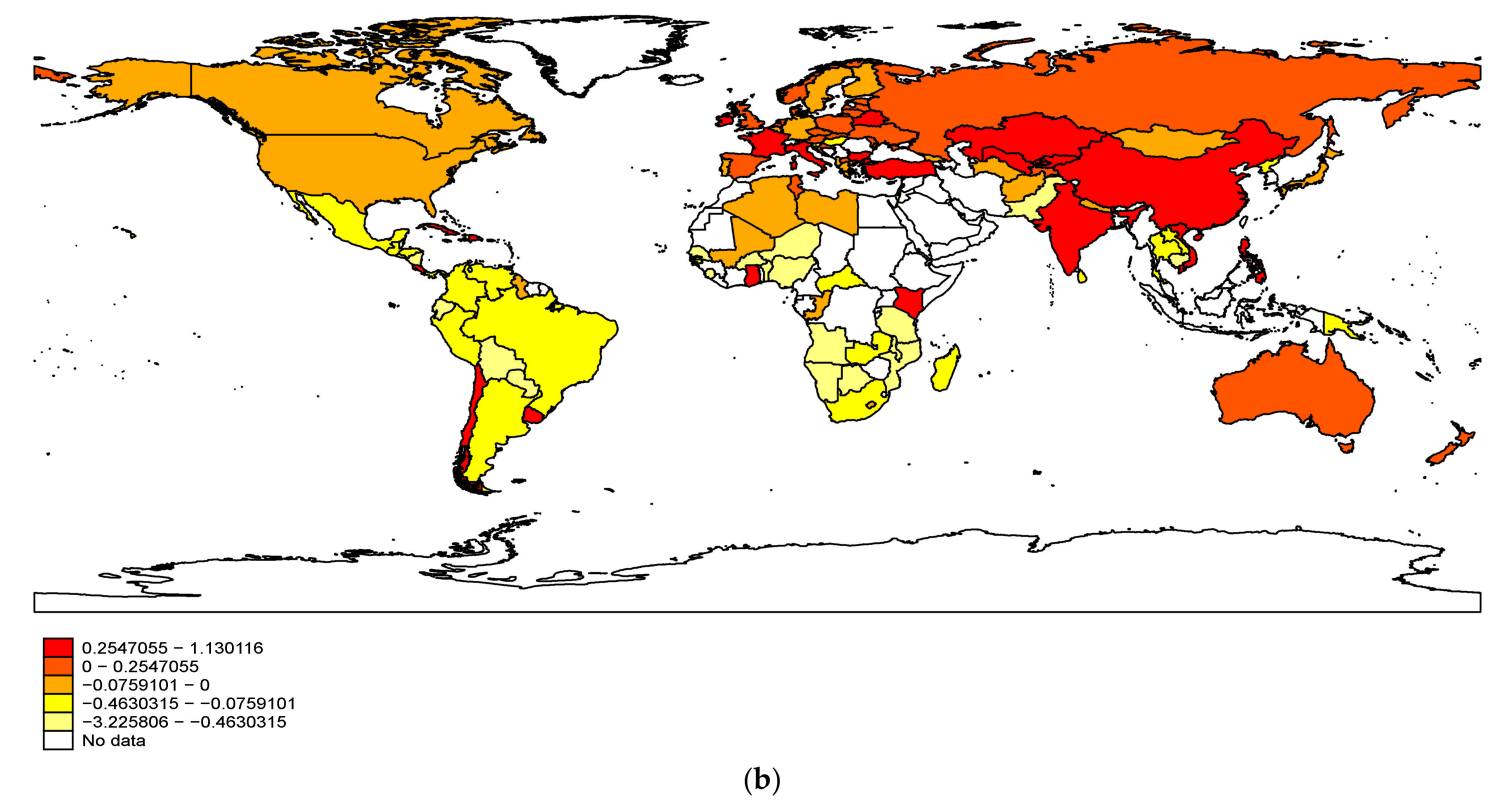
World map of forest growth for countries. (**a**) World map of forest growth in 2001. (**b**) World map of forest growth in 2018. Note: This world map is for the situations of forest growth for sample countries in 2001, which is graphed by using Stata 16 software; the dark color represents higher forest growth. The unit of the value in this map is %. We set 5 intervals to better represent the difference in forest growth among sample countries, we selected the threshold value according to the quintile of the forest growth.

Figure 1 provides the basic distribution of forest protection for the countries in question. We find that China offers the higher forest protection, while Australia and South America show the lowest forest protection.

Globalization (Globalization): while measuring the level of globalization, some scholars utilize international trade or international financial flows to capture the specific dimension of globalization such as trade globalization, financial globalization, and economic globalization (Li et al., 2015; Destek et al., 2018) [36,37]. However, as Khan and Ullah (2019) [38] argued, simply utilizing a variable such as trade or foreign direct investment cannot account the overall impact of globalization on the environment, and may even lead to biased conclusions (Salahuddin et al., 2019; Ulucak et al., 2020) [39,40]. To avoid such problems, according to the research of Salahodjaev (2016) [41] and Feng et al. (2019) [42], we utilized the KOF index of the KOF Swiss Economic Institute updated by Savina et al. (2019) [43] and originally provided by Dreher (2006) [34]; this covers all aspects of globalization such as economic globalization (trade and financial globalization), social globalization (interpersonal, information, and cultural globalization), and political globalization.

According to the existing literature focused on forest growth (Koh and Ghazoul, 2010; Meyfroidt and Lambin, 2011; Hosonuma et al., 2012) [5,16,44], we also include the following variables that may affect forest growth.

Economic performance (GDP): similar to Hao et al. (2019) [45], we utilize real GDP per capita, consistent to 2011, to measure the national economic performance and control the influence of economic performance on forest growth, as denoted by GDP (USD).

Education (Education): in line with Wang et al. (2019) [46], we measure the level of education by the gross secondary school enrollment rate, which is employed to include the influence of education on forest growth.

Urbanization rate (Urban): as suggested by Dale (2018) [47], we use the share of urban residents to total population to measure the urbanization rate, which can capture the potential influence of urbanization on forest growth.

Population (POP): to control the influence of population on forest protection, we measure the population amount by the number of total populations, whose unit is 1 million, denoted by POP (Verma et al., 2021) [48].

Population density (Density): aside from the total populations, we also measure population by density, as higher density may bring about more demand in terms of deforestation in order to offer more space for human living. As provided by Bottero et al., 2017 [49], population density is calculated by the number of people per square km, denoted by Density.

Industrial share (IND): industrial structures increase land use, which may change forest growth. To control the influence of industrial structure on forest growth, we measure industrial structures by the proportion of agricultural value added to GDP (Usman et al., 2021) [50].

Trade openness (Trade): similar to Li et al. (2017a) [21], we utilize the share of exports and imports to GDP to capture the degree of openness, denoted by Trade.

Foreign direct invest (FDI): to measure the producing activities created by foreign direct investment, we include the control variable of FDI as calculated by the ratio of net inward FDI to GDP, which is supported by [51].

Democratic regime (Democracy): to test whether political regimes can change the influence of globalization on forest growth, we include a dummy variable (Democracy) to capture the difference between democratic and autocratic countries. According to the dataset provided by Bjørnskov and Rode (2020) [52], if a country is democratic, Democracy = 1; otherwise, 0.

Share of aging people (Aging): since the aging problem can change a government’s attitude to natural environmental protection, we measure the aging problem by the share of people aged 65 or above in relation to total population (Wang et al., 2021a) [53], denoted by Aging.

Carbon emissions (CO_2_): while governments face worse air quality, they should make greater effort to reduce carbon emissions by improving forest cover. To study the role of air quality in globalization’s influence on forest growth, we measure air quality by carbon dioxide emissions (Estes, 2020) [54], denoted by CO_2_.

Equatorial countries (Equatorial): since forests in equatorial countries are different from other forests in non-equatorial countries, to test whether the influence of globalization on forest growth varies among different kinds of forest, we set a dummy variable (Equatorial) to reflect the difference between equatorial countries and non-equatorial countries (Strindberg et al., 2018) [55].

Emerging markets (EM): according to the classification of the International Monetary Fund and Cuaresma et al. (2017) [56], we set a dummy variable to reflect the characteristics of a country. If the country belongs to an emerging market, then EM = 1; otherwise, 0.

Fiscal freedom (Freedom): as provided by Freedom House, we use fiscal freedom to measure the freedom of a country (denoted by Freedom), which measures the burden of direct as well as indirect taxes on conducting businesses in host countries to investigate whether freedom can change globalization’s impact on forest growth (Busch and Mukherjee, 2018) [57].

Political stability (PS): similar to Galinato and Galinato (2013) [58], we measure the quality of governance for one country by the variable of political stability, which is essential for governments to take advantage of globalization to improve forest coverage. Our definition of political stability is derived from the World Governance Index.

Governmental ideology (Ideology): in line with Chang et al. (2015) [59], we set an encode variable (ideology) to capture the ideology of governments. If a ruling party is left-wing, then ideology = −1; if the ruling party is classified as right-wing, then Ideology = 1; otherwise, 0.

The definitions and data sources of each variable are listed in Table 1. From this table, we find that the data for Forest is obtained from the World Development Indicator (WDI) and Our World in Data; the data for globalization are derived from the KOF Swiss Economic Institute. Data for Democracy can be found in Bjørnskov and Rode (2020) [52], data for Freedom are sourced from the Economic Freedom Index of Freedom House; PS data are from the World Governance Index, data for Ideology are collected from the Database of Political Institute, data for PS is derived from World Governance Index (WGI), and data for other variables are obtained from WDI. After collecting the original data for these variables, we merge them into a panel dataset according to country and year. We hence filter the observations by following principles such as excluding the observations with missing values and the number of those who have fewer than three observations. We finally construct a panel dataset for 108 countries, with a time period ranging from 1991 to 2018. Similar to Wang et al. (2019) [46], we take the log for these variables, except for the variables possessing negative values or the dummy variables. The reasons why we use the logarithm of the variables are as follows: First, the logarithm of the variables will not change the nature and correlation of the data but instead compress the scale of the variables. Second, the logarithm of the variables can better eliminate heteroscedasticity.

The descriptive information of these variables is listed in Table 2. The minimum, mean, median, and maximum of Forest are −4.439, −0.053, −0.010, and 6.678, respectively, and the standard deviation (S.D) is 0.834, suggesting that most countries experience forest loss and that Forest varies among such sample countries. Next, the min and max of Globalization are 3.102 and 4.518, while its mean and S.D are 4.068 and 0.282, supporting the fact that Globalization fluctuates less among sample countries. For other variables, taking GDP and Democracy as examples, we find that the mean and median of GDP are 8.430 and 8.418, while the S.D is 1.475 and the coefficient of variation (CV) is 0.175; this suggests that GDP fluctuates less among such countries. The mean and median of Democracy are 0.719 and 1, with an S.D of 0.450, suggesting that more than 70% of samples are democratic. The statistically descriptive information of other variables can be seen in Table 2.

### 2.2. Econometric Method

According to the traditional econometric theory provided by Wooldridge (2010) [67], panel estimation has several advantages over time-series or cross-section estimation. Specifically, panel estimation takes time and individual dimensions into consideration simultaneously, which can better deal with problems such as missing observations to comprehensively capture dynamic progress. To achieve a reliable conclusion, we also conduct an empirical investigation by employing panel estimation based on multinational panel data. Moreover, forest growth is not only affected by current factors but is determined by previous performances. As such, to include the potential dynamic progress of forest growth and take heterogeneity into account, we utilize the panel system GMM to empirically examine the relationship between globalization and forest growth, which is similar to the approach adopted by Wang et al. (2021b) [22].

(1) $$Forest_{it}=\alpha_{1}Forest_{i,t-1}+\theta Globalization_{it}+\beta^{\prime }X+u_{i}+u_{t}+\varepsilon_{it},$$

In Equation (1), i stands for the individual country; t represents year; $\varepsilon_{it}$ denotes error term. *Forest* is the dependent variable, measured by the change in forest area, whereas $Forest_{i,t-1}$ is the level of forest growth for an earlier term; Globalization is the independent variable, and θ is the corresponding coefficient; if θ is unequal 0 and statistically significant at the given significance level, it supports that Globalization exerts a significant impact at the given significance level; *X* represents the vector of other control variables such as GDP, Education, Urban, POP, Density, IND, Trade, FDI, and Democracy; β is the corresponding coefficient for such a control variable. $u_{i}$ and $u_{t}$ stand for the fixed effect of individual and time, respectively.

The method for estimating Equation (1) is a two-step system, GMM. We utilized software of STATA 16 to conduct systematic GMM estimation; the version of this software is STATA 16 and the computer code for system GMM is available in STATA 16, which is named as “Xtabond2”. 

## 3. Results and Discussion 

### 3.1. Generalized Method of Moment (GMM) Estimations 

Following the method of Wang et al., (2021b) [22], we only include globalization and time effects in the model to examine the influence of globalization on forest growth, thus successively taking into account other control variables. From column 1 in Table 3, one can observe that the coefficient of Globalization is significantly positive at the 1% level with a value of 0.051, implying that countries with higher levels of globalization usually express higher forest growth. Moreover, this idea is confirmed by other results listed in columns 2–6, which incorporate the potential influence of other variables on forest growth. Barring the influence of globalization on forest growth, we can conclude that forest growth is itself a dynamic progress.

These GMM results strongly support a positive link from globalization to forest growth; this may be caused by the awareness of forest growth initiated by environmental globalization, as well as international treaties or NGOs that at to protect forest (Wang et al., 2021b) [22]. Our conclusion is similar to that of Saint Akadiri et al. (2019) [68], who argue that environmental globalization does some good in relation to environmental protection. Our results are also in line with those of Zafar et al. (2019) [69], who propose that globalization could bring about the displacement of national deforestation.

### 3.2. Robustness Tests

To prove our idea that Globalization partially affects forest growth in a positive manner, we carried out several robustness tests.

#### 3.2.1. Change the Measurement of Globalization

First, to avoid the potential bias caused by the measurements, we characterized Globalization as having four specific dimensions: economic, trade, social, and political; these classifications are taken from Rudel (2002) [8] and Grainger (2005) [18]. The GMM estimation employing these four variables can be seen in Table 4, which shows that the coefficient of economic globalization and trade globalization is 0.138 and 0.193, respectively; both are significant at 1%, suggesting that three dimensions of globalization benefit forest growth. However, the coefficients of social globalization and political globalization in columns 3 and 4 are 0.041 and 0.001, respectively, and are insignificant at 10%, supporting the notion that there is no relationship between social or political globalization in terms of forest growth. Our results suggesting that different dimensions of Globalization exert different impacts on forest growth are similar to those elucidated by Wang et al. (2021b) [22], who argue that the various characteristics and influences of specific dimensions of Globalization on the environment need to be investigated. Our results are also similar to those of Li et al. (2017a) [21], who propose that economic globalization creates greater forest cover. The potential reasons for this are as follows. First, economic globalization and trade globalization can reduce domestic non-farm activities through trading abroad (Grau et al., 2013) [24], which can increase forest cover. Next, economic globalization and trade globalization can herald advances in technology (You and Lv, 2018) [70], which can improve production efficiency and change land use to promote forest growth.

#### 3.2.2. Increased or Decreased Globalization

Given global protectionism’s growth since 2017, we include differences related to Globalization in our estimations to examine whether changes in Globalization would influence forest growth. We note that the coefficients of Δ globalization, Δ economic globalization, and Δ trade globalization in Table 5 are 0.086, 0.136, and 0.159, respectively, which are all significant at 5% at least; these results confirm our earlier statement that countries with a higher level of Globalization usually possess higher forest growth. Our results showing that increased Globalization benefits forest growth are in line with the study by Wang et al., (2021b) [22], who argue that accelerating Globalization exerts a positive impact on environmental performance.

#### 3.2.3. Removing the Outliers

To avoid the potential bias caused by outliers, we reconstructed new sub-samples by excluding observations with values smaller than a 10% quantile of forest or those larger than a 10% of forest, which is similar to the approach adopted by Wang et al. (2021a) [53]. The empirical results based on the new samples can be found in Table 6. The coefficients for the four variables of globalization all pass the significance test at 1% with a positive symbol, again confirming our findings.

### 3.3. Moderating Effect of Social Indicators

Once the positive influence of Globalization on forest growth is confirmed, we further query what factors can change Globalization’s impact on forest growth. As Kull et al. (2007) [25] state, while investigating the relationship between globalization and forest, the interventionist effect of other social contexts and ecological factors should be taken into account. Furthermore, Aldyan (2020) [33] proposed that the impact of Globalization might be influenced by economic factors, i.e., the influence of Globalization varies among developed and developing countries. We thus further studies the moderating effect of social, ecological, and economic factors on globalization’s impact on forest growth, such as aging, urbanization, FDI, industrial share, air quality, and equatorial countries.

To begin, we included the cross term Globalization * Aging in our basic equation to test whether the aging problem could change globalization’s impact on forest growth. The coefficient of Globalization * Aging in column 1 of Table 7 is −0.045, which passes the significance test at 1%, suggesting that the relationship between Globalization and forest growth depends on the aging rate. The potential reason for this is that older humans have greater demands regarding air quality and the natural environment, meaning that a serious aging problem exerts higher pressure on governments to protect the forest, weakening the role of Globalization in relation to forests (Zucker, 2013) [71].

Hence, we set the cross term of Globalization * FDI to clarify the moderating effect of FDI on globalization’s impact on forest growth, the result of which is listed in column 2 of Table 7. We found that the coefficient of Globalization * FDI was 0.156, which passes the significance test at 1% and indicates that a greater FDI generates a positive link between globalization and forest growth. This is mainly because the outflow of FDI introduced by Globalization creates more economic activities abroad, which can effectively protect national forests through the displacement of deforestation (Meyfroidt and Lambin 2009) [13]; thus a larger FDI would strengthen the positive impact of Globalization on forest growth.

Next, we examine the role of industrial share on Globalization’s influence on forest growth by setting the cross term Globalization * IND, the result of which is given in column 3 of Table 7. We note that the coefficient of Globalization * IND is −0.079, which passes the significance test at 1%, confirming that a higher share of industry would weaken the correlation between Globalization and forest growth. The potential reason for this may be that, if the share of industry is higher, the economy might shift from agriculture to industry, which benefits forest recovery and reduces opportunities to protect forests and the environment that Globalization provides (Wang et al., 2020) [72].

Moreover, to test the moderating effect of air quality on globalization’s positive impact on forest growth, we include the cross term Globalization * CO_2_ in our estimation, the result of which is listed in column 4 of Table 7. It can be found that the coefficient of Globalization * CO_2_ is −0.04, which passes the significance test at the 1% level, implying that higher CO_2_ emissions inhibit the positive correlation between Globalization and forest growth. The potential reason for this is that one of the adverse effects of Globalization is environmental damage, such as CO_2_ emissions (Khan and Ullah, 2019; Pata, 2021) [38,73]. Higher CO_2_ emissions led by Globalization suggest that it could cause adverse effects, thus reducing the impact of globalization on forest growth.

Additionally, considering the particularity of tropical forests, we also test whether the influence of globalization on forest growth varies between equatorial countries and non-equatorial countries by setting the cross term Globalization * Equatorial, as provided in column 5 of Table 7. The coefficient of Globalization * Equatorial is 1.548, which is positive but not significant, indicating that the relationship between Globalization and forest growth is constant between equatorial countries and non-equatorial countries.

Finally, we test whether the influence of Globalization on forest growth varies between emerging markets and non-emerging markets by including the cross term Globalization * EM in our model, the result of which is listed in column 6 of Table 7. The coefficient of Globalization * EM is −0.683, which passes the significance test at the 1% level, suggesting that emerging markets possess weaker relationships between Globalization and forest growth. This is primarily because developing countries are highly influenced by economic globalization, which causes adverse effects on the environment and thus leads to the destruction of forests (Aldyan, 2020) [33].

Our results suggest that social and economic factors such as aging rate, FDI, industrial structure, air quality, and emerging markets affect the relationship between Globalization and forest growth, which is similar to the results attained by Kull et al. (2007) [25], who propose that the impact of Globalization on national performance depends social and economic indicators; our findings are also similar to those of Aldyan (2020) [33], who suggest that economic development may change Globalization’s impact on forests.

### 3.4. The Impact of Different Political Regimes

Aside from social or economic indicators, the influence of globalization on forest growth also varies among different political regimes (Kull et al., 2007) [25]. Forest management is influenced by corruption, business activities, institutional quality, and policies (Langner and Siegert, 2007) [74]. Furthermore, in the era of globalization, in which environmental pollution is a known global issue, international democracy is beneficial for countries in terms of sharing updated technologies and information regarding environmental issues to promote the development of international treaties to resolve global environmental problems (Winslow, 2005) [75]. To comprehensively study whether the influence of globalization on forest growth varies between different political regimes, we examine political characteristics from the perspectives of democracy, quality of governance, governmental ideology, and political stability.

At first, we examine whether globalization’s influence on forest growth is similar between democratic and autocratic regimes by setting the cross term Globalization * Democracy, the result of which is listed in column 1 of Table 8. We find that the coefficient of Globalization * Democracy is −0.080 and is significantly negative at 5%, implying that, among democracies, the positive influence of globalization on forest growth is weaker. A potential reason for this is that international democracy is beneficial for countries being able to share more updated technologies and information regarding environmental issues (Winslow, 2005) [75], which may weaken the role of globalization.

Furthermore, we further examine whether the quality of governance can change Globalization’s influence on forest growth via the cross term Globalization * Freedom. As displayed in column 2, the coefficient of Globalization* Freedom is −0.008, which is significantly negative at the 1%, supporting the notion that fiscal freedom would reduce the positive influence of globalization on forest growth. This is mainly because forest degradation is related to business activities; higher levels of freedom may potentially create more activities (Russell et al., 2020) [76], especially investment abroad, which may cause the loss of forests and reduce the positive impact of Globalization on forest growth (Armenteras et al., 2017) [77].

In addition, we further examine whether globalization’s influence on forest growth varies between left- or right-wing governments by including the cross term Globalization * Ideology, as provided in column 3. The coefficient of Globalization * Ideology is −0.039, which is insignificant at the 10% level, indicating that governmental ideology cannot change globalization’s role in forest growth.

Finally, we test whether political stability determines globalization’s influence on forest growth via the cross term Globalization * PS, the result of which is listed in column 5. We observe that the coefficient of Globalization * PS is 0.071, which is significantly positive at 1%, implying that, when political stability is higher, the positive link from globalization to forest growth is stronger. This is mainly because higher political stability means that governments can better allocate fiscal resources in the long term and effectively conduct measures to protect forest growth (Galinato and Galinato, 2013) [58]; this provides them with greater opportunities to take advantage of Globalization to promote forest growth (Grima and Singh, 2019) [78].

## 4. Conclusions

Given the growth of protectionism in recent years, this paper attempts to uncover the relationship between globalization and the natural environment from the perspective of forest growth. Our baseline result indicates that globalization promotes the protection of forests. Moreover, social and political globalization cannot change forest growth, while the other three dimensions of globalization benefit it. Furthermore, if the rate of globalization is high, forest growth experiences an increase. In addition, the benefit of FDI in terms of globalization’s positive impact on forest growth is important to consider; a higher aging rate, industrial share, CO_2_ emissions, and business freedom weaken the influence of globalization on forest growth, and the influence of globalization on forest growth is weaker among emerging markets. Finally, compared to autocracies, democracies are more likely to take advantage of globalization to promote forest growth; similarly, the influence of globalization on forest growth is weaker among countries with higher levels of freedom or lower levels of political instability. However, globalization’s positive impact on forest growth is consistent between left- and right-wing countries.

By reviewing the existing literature, we can firmly argue that this study is the first to investigate the moderating effect of social indicators on globalization’s impact on forests, as well as the first to test whether the influence of globalization on forest growth varies among different political regimes. As such, this study is novel in this field.

Based on our empirical results, we provide the following policy implications for governments to better protect forests. First, it is necessary for policymakers to promote the progress of environmental globalization and continue employing open measures of globalization, such as increasing the import of agriculture products, which can protect national forests through the displacement of deforestation. Second, since cultural globalization can also help governments achieve greater forest growth, governments should make more efforts to spread a worldwide culture that positively affects social development, thus improving awareness about forest growth.

It is worth noting that our results for a moderating effect support the notion that globalization exerts a positive impact on forest growth among countries with a greater FDI. Thus, local governments should carry out feasible policies such as tax incentives or subsidies, as well as measures to protect investors overseas, thus promoting the investment of more capital abroad, which can improve national forests through global activities. In addition, emerging markets reduce the positive role of globalization on forest growth. For emerging markets, while they take the advantage of globalization to spur their economic development, the ecosystem and natural environment should not be ignored. While they attract the overseas investments, regulations of environmental protection or forest growth should be carried out. In some case, governments can refuse investors who engage in activities that cause serious environmental damage.

## Figures and Tables

**Table 1:** Variable definition.

Variables	Definition	Source
Dependent variables		
Forest	The proportion of forest growth to earlier forest area	WDI (2020) [60]; Ritchie and Roser (2020) [61]
Independent Variables		
Globalization	The KOF globalization index	Savina et al. (2019) [43]
Control variables		
GDP	National per capita real GDP in constant 2011 US dollars	WDI (2020) [60]
Education	The gross secondary school enrollment rate	WDI (2020) [60]
Urban	The share of urban residents in total population	WDI (2020) [60]
POP	The number of total populations, whose unit is million	WDI (2020) [60]
Density	The number of people per square km	WDI (2020) [60]
IND	The proportion of agriculture value added to GDP.	WDI (2020) [60]
Trade	Share of exports and imports to GDP	WDI (2020) [60]
FDI	Ratio of net inflow of FDI (IFDI) to GDP	WDI (2020) [60]
Democracy	Dummy variable to capture the democratic or autocratic regime	Bjørnskov and Rode (2020) [52]
Aging	The share of people aging 65 or above to the total populations	WDI (2020) [60]
CO_2_	The total amount of carbon emissions, whose unit is million tons	WDI (2020) [60]
Equatorial	Dummy variable to measure the equatorial one	Demarty and Bastien (2011) [62]
EM	Dummy variable to measure the Emerging Markets	Long and Ascent (2020) [63]
Freedom	The burden of direct as well as indirect taxes on conducting businesses in the host countries	Freedom House (2020) [64]
PS	The degree of political stability	WGI (2020) [65]
Ideology	An encode variable to measure the ideology of government	Cruz et al. (2021) [66]

Note: the GDP stands for the gross domestic products, FDI represents the foreign direct investment, WDI denotes for World Development Indicator, and WGI means World Governance Index.

**Table 2:** Basic descriptive statistics of variables.

Variable	Observations	Min	Mean	Median	S. D.	Max	CV
Forest	2736	−4.439	−0.053	−0.010	0.834	6.678	−15.723
Globalization	2736	3.102	4.068	4.097	0.282	4.518	0.069
GDP	2729	5.306	8.430	8.418	1.475	11.425	0.175
Education	1984	1.838	4.291	4.500	0.563	5.088	0.131
Urban	2736	2.359	3.956	4.073	0.461	4.594	0.117
POP	2736	12.707	16.313	16.137	1.498	21.055	0.092
Density	2736	0.894	4.015	4.196	1.256	6.508	0.313
IND	2461	0.000	2.629	2.704	0.447	3.933	0.170
Trade	2736	2.756	4.232	4.216	0.471	5.620	0.111
FDI	2459	−5.116	0.535	0.298	0.823	5.711	1.539
Democracy	2709	0.000	0.719	1.000	0.450	1.000	0.625
Aging	2736	1.932	8.424	6.161	5.540	27.576	0.658
CO_2_	2544	0.191	4.233	4.111	1.673	9.358	0.395
Equatorial	2736	0.000	0.039	0.000	0.195	1.000	4.934
EM	2736	0.000	0.181	0.000	0.385	1.000	2.126
Freedom	2375	0.000	71.767	74.540	22.825	87.300	0.318
PS	2059	−2.810	−0.071	−0.019	0.881	1.760	−12.484
Ideology	2012	−1.000	0.158	1.000	0.926	1.000	5.861

**Table 3:** The impact of globalization on forest growth.

	(1)	(2)	(3)	(4)	(5)	(6)
L. Forest	0.968 ***	0.958 ***	0.942 ***	0.940 ***	0.916 ***	0.863 ***
	(183.245)	(164.019)	(72.163)	(76.602)	(65.433)	(54.073)
Globalization	0.051 ***	0.113 ***	0.162 ***	0.171 ***	0.122 ***	0.265 ***
	(6.001)	(6.145)	(4.827)	(5.344)	(4.000)	(5.280)
GDP		−0.011 ***	0.009	0.010 *	−0.001	−0.032 **
		(−4.735)	(1.639)	(1.759)	(−0.164)	(−2.314)
Education			−0.042 ***	−0.049 ***	−0.023 *	−0.039 *
			(−4.096)	(−4.614)	(−1.706)	(−1.892)
Urban			−0.042 ***	−0.029 ***	0.037	0.080 **
			(−3.423)	(−2.695)	(1.614)	(2.554)
POP				−0.002	0.006 *	0.001
				(−0.594)	(1.682)	(0.239)
Density				−0.004	0.001	0.022 *
				(−0.476)	(0.054)	(1.646)
IND					−0.036 ***	−0.059 ***
					(−3.899)	(−5.077)
Trade					0.018 *	0.003
					(1.718)	(0.180)
FDI						0.011 ***
						(4.067)
Democracy						0.027 ***
						(3.036)
Year	yes	yes	yes	yes	yes	yes
Cons	−0.211 ***	−0.379 ***	−0.414 ***	−0.426 ***	−0.660 ***	−1.000 ***
	(−6.307)	(−6.352)	(−3.976)	(−4.066)	(−5.050)	(−5.507)
N	2736	2729	1984	1984	1824	1684
AR (1)-P	0.002	0.002	0.011	0.011	0.012	0.012
AR (2)-P	0.131	0.104	0.148	0.160	0.182	0.220
Hansen	46.12	45.25	47.15	47.19	34.44	39.46
Hansen-P	0.998	0.999	0.992	0.987	1.000	0.998

Notes: The statistics in parenthesis are the value of Z-statistics for corresponding coefficient. * *p* < 0.1, ** *p* < 0.05, and *** *p* < 0.01.

**Table 4:** Robustness test—changing the measurement of Globalization.

	(1)	(2)	(3)	(4)
L. Forest	0.866 ***	0.854 ***	0.875 ***	0.864 ***
	(59.030)	(44.538)	(57.665)	(61.181)
Economic Globalization	0.138 ***			
	(3.563)			
Trade Globalization		0.193 ***		
		(5.371)		
Social Globalization			0.041	
			(0.908)	
Political Globalization				0.001
				(0.994)
GDP	−0.008	−0.009	−0.005	−0.014
	(−0.641)	(−0.680)	(−0.508)	(−1.470)
Education	−0.001	−0.011	−0.040 *	−0.019
	(−0.035)	(−0.442)	(−1.928)	(−1.122)
Urban	0.004	0.022	0.090 **	0.108 ***
	(0.132)	(0.682)	(2.477)	(3.769)
POP	0.006	−0.000	0.006	0.005
	(1.049)	(−0.021)	(1.312)	(1.054)
Density	0.024 **	0.028 **	0.029 **	0.032 ***
	(2.322)	(2.299)	(2.317)	(2.648)
IND	−0.047 ***	−0.058 ***	−0.066 ***	−0.069 ***
	(−4.566)	(−4.853)	(−5.686)	(−5.766)
Trade	−0.019	−0.077 ***	0.028 *	0.036 **
	(−1.003)	(−3.482)	(1.834)	(2.477)
FDI	0.006 **	0.009 **	0.014 ***	0.014 ***
	(2.361)	(1.991)	(4.681)	(6.271)
Democracy	0.048 ***	0.028 ***	0.033 ***	0.031 ***
	(8.771)	(3.063)	(3.388)	(3.588)
Year	yes	yes	yes	yes
Cons	−0.539 ***	−0.408 ***	−0.541 ***	−0.500 ***
	(−2.994)	(−2.728)	(−3.658)	(−4.267)
N	1684	1684	1684	1684
AR (1)-P	0.013	0.012	0.012	0.013
AR (2)-P	0.190	0.394	0.651	0.506
Hansen	35.24	38.54	42.14	44.17
Hansen-P	1.000	0.998	0.994	0.989

Notes: The statistics in parenthesis are the value of Z-statistics for corresponding coefficient. * *p* < 0.1, ** *p* < 0.05, and *** *p* < 0.01.

**Table 5:** The impact of Globalization’s change on forest growth.

	(1)	(2)	(3)
L. Forest	0.856 ***	0.870 ***	0.867 ***
	(54.863)	(55.312)	(46.202)
ΔGlobalization	0.086 **		
	(2.301)		
Δ Economic Globalization		0.136 ***	
		(4.686)	
Δ Trade Globalization			0.159 ***
			(5.436)
GDP	−0.005	0.003	−0.005
	(−0.573)	(0.270)	(−0.407)
Education	−0.017	−0.036 *	−0.022
	(−0.760)	(−1.917)	(−0.894)
Urban	0.100 ***	0.075 ***	0.098 ***
	(3.138)	(2.942)	(3.236)
POP	0.007	0.006	0.005
	(1.581)	(0.985)	(0.833)
Density	0.030 ***	0.032 **	0.035 **
	(2.646)	(2.234)	(2.576)
IND	−0.070 ***	−0.053 ***	−0.075 ***
	(−5.845)	(−4.351)	(−5.880)
Trade	0.040 **	0.017	0.020
	(2.392)	(0.915)	(0.927)
FDI	0.012 ***	0.010 ***	0.012 ***
	(4.792)	(2.987)	(2.745)
Democracy	0.025 ***	0.034 ***	0.028 ***
	(2.822)	(5.293)	(2.892)
Year	yes	yes	yes
Cons	−0.557 ***	−0.375 **	−0.414 ***
	(−3.930)	(−2.472)	(−2.716)
N	1684	1684	1684
AR (1)-P	0.013	0.012	0.012
AR (2)-P	0.337	0.198	0.344
Hansen	45.49	41.89	43.78
Hansen-P	0.984	0.995	0.990

Notes: The statistics in parenthesis are the value of Z-statistics for corresponding coefficient. * *p* < 0.1, ** *p* < 0.05, and *** *p* < 0.01.

**Table 6:** Removing the outliers.

	(1)	(2)	(3)	(4)
L. Forest	0.907 ***	0.900 ***	0.878 ***	0.908 ***
	(49.256)	(46.807)	(52.824)	(41.057)
Globalization	0.132 ***			
	(3.249)			
Economic Globalization		0.236 ***		
		(8.331)		
Trade Globalization			0.132 ***	
			(5.401)	
Culture Globalization				0.069 ***
				(2.874)
GDP	−0.010	−0.014	−0.006	−0.011
	(−1.084)	(−1.390)	(−0.692)	(−1.010)
Education	−0.040 ***	−0.034 **	−0.019 *	−0.055 ***
	(−3.332)	(−2.488)	(−1.721)	(−3.993)
Urban	0.017	−0.039 *	−0.014	0.015
	(0.754)	(−1.700)	(−0.844)	(0.781)
POP	0.001	0.005	0.000	−0.004
	(0.200)	(1.106)	(0.022)	(−0.852)
Density	0.028 ***	0.018 **	0.025 ***	0.030 ***
	(3.371)	(1.988)	(3.079)	(3.064)
IND	−0.032 ***	0.002	−0.012	−0.017 ***
	(−4.538)	(0.241)	(−1.513)	(−3.094)
Trade	0.002	−0.051 ***	−0.050 ***	−0.001
	(0.233)	(−4.303)	(−3.545)	(−0.101)
FDI	−0.011 ***	−0.012 ***	−0.006 ***	−0.006 **
	(−4.341)	(−4.748)	(−2.695)	(−2.425)
Democracy	0.087 ***	0.088 ***	0.087 ***	0.093 ***
	(10.532)	(11.302)	(13.062)	(13.784)
Year	yes	yes	yes	yes
Cons	−0.485 ***	−0.545 ***	−0.263 ***	−0.088
	(−3.895)	(−4.955)	(−2.785)	(−0.870)
N	1316	1316	1316	1316
AR (1)-P	0.000	0.000	0.000	0.000
AR (2)-P	0.594	0.725	0.921	0.933
Hansen	38.73	36.02	32.29	36.34
Hansen-P	0.996	0.999	1.000	0.998

Notes: The statistics in parenthesis are the value of Z-statistics for corresponding coefficient. * *p* < 0.1, ** *p* < 0.05, and *** *p* < 0.01.

**Table 7:** Generalized method of moment (GMM) estimation for moderating effect-social and geographical feature.

	(1)	(2)	(3)	(4)	(5)	(6)
L. Forest	0.790 ***	0.872 ***	0.863 ***	0.852 ***	0.868 ***	0.865 ***
	(40.965)	(41.949)	(53.384)	(44.958)	(47.611)	(44.052)
Globalization	0.329 ***	0.247 ***	0.430 ***	0.397 ***	0.162 ***	0.183 ***
	(2.986)	(2.621)	(5.449)	(5.433)	(2.755)	(3.333)
Globalization*Aging	−0.045 ***					
	(−3.244)					
Aging	0.214 ***					
	(3.610)					
Globalization*FDI		0.156 ***				
		(4.207)				
Globalization*IND			−0.079 ***			
			(−2.603)			
Globalization*CO_2_				−0.040 ***		
				(−2.576)		
CO_2_				0.172 ***		
				(2.600)		
Globalization*Equatorial					1.548	
					(0.869)	
Equatorial					−6.462	
					(−0.899)	
Globalization*EM						−0.683 ***
						(−3.381)
EM						2.902 ***
						(3.366)
Control/Year	yes	yes	yes	yes	yes	yes
Cons	−1.190 ***	−0.938 ***	−1.746 ***	−1.301 ***	−0.909 ***	−0.622 *
	(−3.420)	(−3.319)	(−4.809)	(−5.569)	(−4.068)	(−1.891)
N	1684	1684	1684	1684	1684	1684
AR (1)-P	0.013	0.010	0.012	0.012	0.012	0.011
AR (2)-P	0.226	0.908	0.258	0.534	0.172	0.157
Hansen	27.04	36.90	38.02	33.43	28.78	30.62
Hansen-P	1.000	0.999	0.998	1.000	1.000	1.000

Notes: The statistics in parenthesis are the value of Z-statistics for corresponding coefficient. * *p* < 0.1, and *** *p* < 0.01.

**Table 8:** Moderating effect of political indicators.

	(1)	(2)	(3)	(4)
L. Forest	0.853 ***	0.745 ***	0.813 ***	0.854 ***
	(52.831)	(26.865)	(41.238)	(27.127)
Globalization	0.320 ***	1.106 ***	0.284 ***	0.350 **
	(5.921)	(3.606)	(2.841)	(2.299)
Globalization*Democracy	−0.080 **			
	(−2.034)			
Globalization* Freedom		−0.008 ***		
		(−2.843)		
Freedom		0.036 ***		
		(2.907)		
Globalization*Ideology			−0.039	
			(−1.264)	
Ideology			0.194	
			(1.506)	
Globalization*PS				0.071 **
				(2.090)
PS				−0.271 **
				(−2.110)
Democracy	0.330 **	−0.067 ***	0.017	−0.004
	(2.086)	(−4.331)	(0.666)	(−0.327)
Control/Year	yes	yes	yes	yes
N	1684	1537	1314	1331
AR (1)-P	0.012	0.012	0.045	0.097
AR (2)-P	0.240	0.948	0.173	0.111
Hansen	41.76	35.72	33.93	17.04
Hansen-P	0.993	0.984	0.999	0.997

Notes: The statistics in parenthesis are the value of Z-statistics for corresponding coefficient. ** *p* < 0.05, and *** *p* < 0.01.

## Data Availability

The data used to support the findings of this study are available from the corresponding author upon request.
